# Major Components of Metabolic Parameters and Nutritional Intakes in Different Genotypes of Adiponectin +276 G>T Gene Polymorphism in Non-Diabetes and Non-Alcoholic Iranian Fatty Liver Patients

**Published:** 2017

**Authors:** Fatemeh Mohseni, Sahar Moghbelinejad, Reza Najafipour

**Affiliations:** Cellular and Molecular Research Center of Qazvin University of Medical Science, Qazvin, Iran

**Keywords:** Adiponectin, Nonalcoholic fatty liver disease (NAFLD), Polymorphism

## Abstract

**Background:**

Genetic and environmental factors are both involved in the etiology of Non-Alcoholic Fatty Liver Disease (NAFLD). Among the genetic factors, certain polymorphisms of adiponectin gene are associated with NAFLD. In the current study, we investigated the association between metabolic parameters with different genotypes of adiponectin +276 G>T polymorphism among the Iranian NAFLD patients, and the effect of nutritional intake with development of NAFLD.

**Methods:**

In this study, 75 patients with NAFLD and 76 healthy individuals were enrolled. Dietary intakes were assessed using a semi-quantitative Food-Frequency Questionnaire (FFQ). Body Mass Index (BMI) and Waist to Hip Ratio (WHR) were calculated. Biochemical assays including FSG (Fasting Serum Glucose), liver enzymes, lipid profiles, Malondialdehyde, insulin resistance and Total Antioxidant Capacity (TAC) were measured after 12 *hr* fasting. Gene polymorphism study was done by using of sequencing method.

**Results:**

Although, T allele frequency was more prevalent in patients with NAFLD than control, adiponectin +276 G>T polymorphism was not associated with risk of NAFLD. Among the metabolic parameters, TAC in TT genotype was significantly lower 1.44(0.69 to 2.81) p>0.05, AST in GT, GG genotypes, and ALT in all three genotypes were higher in NAFLD patients in compared to healthy subjects (p<0.05). Patients with GT genotype have significantly lower fat consumption and vitamin E intake as compared to control group with the same genotype (p<0.05).

**Conclusion:**

In this study, we showed the association of different genotypes of +276 G>T polymorphism in adiponectin gene with some metabolic parameters.

## Introduction

Non-Alcoholic Fatty Liver Disease (NAFLD) is one of the most common hepatic disorders with unusual lipid deposition in hepatocytes ^1^. NAFLD is an epidemic metabolic liver disease observed in many countries and its prevalence is increasing worldwide ^2^. According to the US National Health and Nutrition Examination Survey (NHANES), the prevalence of NAFLD among chronic liver diseases grew from 47 to 75% between 1988 and 2008 ^1^. The prevalence of NAFLD in the adult general population was reported 21.5% in a large population-based study in southern regions of Iran in 2011 ^3^.

NAFLD is the hepatic manifestation of the metabolic syndrome because obesity and insulin resistance are in close inter-relationship with NAFLD ^1,4^. Numerous studies have suggested that insulin resistance is characteristic of NAFLD ^5^. Insulin resistant patients with NAFLD show decreased insulin sensitivity either at the level of muscle or at the level of liver and adipose tissue ^6,7^. Insulin resistance is related to increase of Free Fatty Acids (FFAs) flux that increases TG production and secretion of Very Low-Density Lipoprotein (VLDL) is stimulated in hepatocytes. Fat accumulation in liver is linked with lipid peroxidation and oxidative stress.^5^ Oxidative stress phenomenon induces imbalance in the pro-oxidant/antioxidant equilibrium; a condition that may influence a number of pathophysiological events in the liver ^8^.

Genetic and environmental factors are both involved in etiology of NAFLD as a multifactorial disease; genetic polymorphisms and dietary intake have been identified as influencing factors in NAFLD development ^9^. The long-term excessive intake of dietary composition in food groups is associated with NAFLD progression ^10^. Generally, lower antioxidant consumption, higher intake of energy, protein and carbohydrate stimulate hepatic lipid accumulation leading to development of fatty liver disease ^11,12^. Some studies mostly in animal models introduced diet as a potent modifier of NAFLD-related genes expression ^10^. An obvious genetic participation to NAFLD is estimated as 39% ^13^. The gene coding for adiponectin is located on chromosome 3q27 including 3 exons and 2 introns and contains 244 amino acids ^2^.

Adiponectin protein is involved in regulating glucose levels as well as fatty acid breakdown. Reduction in levels of adiponectin which is produced by adipocytes has been reported in several studies in patients with insulin resistance, metabolic syndrome, obesity and NAFLD ^2,14–17^. One of the most common Single Nucleotide Polymorphisms (SNPs) of adiponectin gene is rs1501299 (+276 G>T) in the intronic region which has been reported by some epidemiological studies ^2,18^. This variant (rs1501299) is associated with low adiponectin expression and it might be related to overweight and insulin resistance ^2^. In this research, our aim was to evaluate the, association of different genotypes of +276 G>T polymorphism in adiponectin gene with some metabolic parameters, and nutritional intake with development of NAFLD.

## Materials and Methods

### Subjects

The present case-control study was performed among 151 volunteers aged 20–50 with Body Mass Index (BMI) between 25 and 39.9 *kg/m*^2^ including 75 patients with NAFLD and 76 healthy individuals; these two groups were matched by age and gender with group matching. Diagnosis was confirmed by the physician based on the findings of ultrasonography. The patients had simple steatosis (grade 1–2). Control group was composed of the volunteers from university staff and relatives of patients. They were matched by age and gender with case group. Also ultrasonography was done for healthy volunteers to confirm the absence of NAFLD. Written informed consent was obtained from all of participants.

Patients with acute or chronic liver diseases, viral hepatitis, hemochromatosis, Wilson’s disease, autoimmune or endocrine disorders, pregnancy or lactation, alcohol consumption, use of hepatotoxic medications and being on weight loss diets for at least 3 months prior to participation in study were excluded. These criteria were asked from volunteers; however some of them had valid medical history. Written informed consent was obtained from all subjects before entering the study.

### Anthropometric assessments

Weight of the subjects was measured with a calibrated scale (SECA, Hamburg, Germany) with the precision of nearest 0.1 *kg*, while they were in minimal clothing without shoes. Height was measured by non-stretchable measurement tape with the precision of 0.1 *cm*. The BMI was calculated as weight (*Kg*) divided by height squared (*m*^2^). Waist Circumference (WC) was carried out in standing position at the level of the umbilicus and Hip Circumference (HC) was measured at the maximum circumference between the hip and the buttock with a non-elastic tape.

### Biochemical assessments

After an overnight fast, 7 *ml* venous blood samples were obtained from subjects. Approximately 2 *ml* of the blood was transferred into tubes containing Ethylene Diamine Tetra Acetic acid (EDTA) for genetic assays. Serum samples were extracted for biochemical assays from remaining blood. Measuring of Fasting Serum Glucose (FSG), Alanine aminotransferase (ALT), Aspartate aminotransferase (AST), Total Cholesterol (TC), Triglyceride (TG) and High Density Lipoprotein cholesterol (HDL-C) were assessed by Abbott ALCYON ^TM^ 300 auto analyzer using commercial ELISA kits (Pars-Azmoon, Tehran, Iran). Serum LDL-C was calculated by Friedewald formula ^19^. Insulin resistance was estimated by the homeostasis model (HOMA-IR) by following equation: fasting serum insulin (*µU/ml*)×fasting glucose (*mg/dl*)/405 ^20^. Measuring of serum Total Antioxidant Capacity (TAC) was performed by colorimetric method using Randox Kit (Randox laboratories ltd., UK). Malondialdehyde (MDA) levels were measured by Thiobarbituric Acid Reactive Substances (TBARS) method. Samples were heated with 0.6% thriobarbituric acid under acidic condition; the colored product was extracted into n-butanol after cooling. The color absorbance was measured at 530 *nm*. MDA standards were made with 1, 1, 3, 3 – tetraethoxypropane. All of the biochemical assays were conducted by a trained lab assistant who was blinded to group assignments.

### Dietary intake

Dietary intakes were assessed using a semi-quantitative Food-Frequency Questionnaire (FFQ) adapted to the Iranian society ^21^. The FFQ included 168 food items with specified serving sizes commonly consumed by Iranians. Participants reported their average frequency intake of each food item during the previous year in terms of the number of specified serving sizes consumed per day/week/month/year, or never. The reported frequency of consumed foods and portion sizes for each food item were converted to a daily intake.

### DNA extraction and genotyping

Genomic DNA was isolated from the blood cells by salting out method ^22^. DNA fragment analogous to the polymorphisms of +45T>G (rs2241766) and +276G>T (rs1501299) were amplified by primers, 5’-ATCAAG GTGGGCTGCAATA-3’ as forward primer and 5’-TGGGAATAGGGATGAGGGT-3’ as reverse primer, respectively. For doing Polymerase Chain Reaction (PCR), 1 *µl* of genomic DNA, 0.2 *µl* of Taq DNA polymerase and 1 *µl* from each primers were added to 22 *µl* of 1×PCR master-mix. PCR procedure included a primary denaturation at 95*ºC* for 2 *min* followed by 30 cycles of denaturation at 95*ºC* for 30 *s*, annealing at 61.3*ºC* for 30 *s*, extension at 72*ºC* for 20 *s* and a final extension at 72*ºC* for 5 *min*. Sequencing of PCR products (PCR products were directly used for sequencing) was carried out according to Sanger method using ABI 3730XL Capillary Sequencer. Sequencing results were compared with the sequence of normal adiponectin gene obtained from NCBI website: http://www.ncbi.nlm.nih.gov; also, sequence traces were assembled using Chromas software (version 2.4).

### Statistical analysis

All data analysis was performed using the SPSS software (Statistical Package for the Social Sciences, SPSS Inc., Chicago, IL, USA). The normality of variables was assessed by Kolmogorov-Smirnov test. Variables are expressed as means±Standard Deviation (SD) or numbers and percentages. The comparison of continuous variables between two groups was performed by independent sample t-test. Comparison of continuous variables between different genotypes was performed by Analysis of Covariance (ANCOVA) with adjustment for the confounder effect of age and sex. Categorical variables were also compared using the *χ*^2^ test. Logistic regression analysis was used to assess the relationship between anthropometric and biochemical variables with NAFLD genotypes adjusted for the confounding role of sex and age. p-value of less than 0.05 was considered to be significant.

## Results

The demographic characteristics are presented in table 1. WC and WHR in NAFLD patients were significantly higher as compared to healthy group (p<0.05). Serum HDL-C and LDL-C concentrations were lower and serum AST, ALT and TG concentrations were higher in NAFLD patients compared with control group (p<0.05 and p<0.01, respectively) shown in table 2. The genotype and allele frequencies of adiponectin +276 G>T gene polymorphism are shown in table 3. In NAFLD patients at position +276 of adiponectin gene, (44.0%), (42.7%) and (13.3%) subjects had GG, GT and TT genotype, respectively; in control group these frequencies were GG (51.3%), GT(36.8%), TT(11.8%). However, significant statistical difference was not achieved between groups. In accordance to our finding, T allele frequency was more prevalent in patients with NAFLD than in control, but this high frequency was not significant (p>0.05), (Table 3).

**Table 1.** Demographic characteristics of study subjects

**Variable**	**NAFLD (n =75) n(%)**	**Control (n=76) n(%)**	**Mean Difference (95%CI)**	p ^*^
**Gender**				
Male n (%)	36 (48%)	29 (38.2%)	-	0.252
Female n (%)	39 (52%)	47 (61.8%)	-	
**Age (years)**	40.65(8.41)	38.87(8.2)	1.78(−0.89 to 4.46)	0.189
**BMI (*kg/m*^2^)**	31.78 (4.17)	31.38(4.04)	0.40(−0.92 to 1.72)	0.549
**WC (*cm*)**	103.12(9.46)	100.14(8.72)	2.98(0.55 to 5.90)	**0.046**
**WHR**	0.92(0.06)	0.89(0.06)	0.02(0.002 to 0.04)	**0.031**

BMI, body mass index; WC, waist circumference; WHR, waist to hip ratio.

* p-value for gender based on Chi-Square Tests and p-value for other variables based on 2-tailed independent T-test using equal variable.

**Table 2.** Biochemical parameters of study subjects

**Variable**	**NAFLD (n=5) mean (SD)**	**Control (n=76) mean (SD)**	**Mean Difference (95%CI)**	**p ^†^**
**TC (*mg/dl*)**	183.44(36.91)	187.96(28.89)	−4.52(−15.17 to 6.13)	0.403
**HDL (*mg/dl*)**	43.24(11.4)	48.29(11.6)	−5.05(−8.75 to −1.35)	**0.008**
**LDL (*mg/dl*)**	104.11(34.62)	111.52(26.43)	−6.81(−16.71 to 3.09)	**0.030**
**FSG (*mg/dl*)**	90.59(11.24)	89.59(9.93)	0.63(−2.78 to 4.03)	0.717
**ALT (*IU/l*)**	49.96(25.958)	26.84(9.814)	23.12(16.82 to 29.41)	**<0.001**
**AST (*IU/l*)**	32.99(14.86)	23.08(6.12)	9.91(6.26 to 13.55)	**<0.001**
TG (*mg/dl*) ^*^	152.00(114.00–225.00)	118.50(79.50–198.00)	-	**0.004**
**MDA (*nmol/ml*)**	2.82±1.01	2.75±1.03	−0.056(−0.38–0.27)	0.734
**TAC (*µm/L*)**	1.45±0.26	1.58±0.28	0.12(0.03–0.21)	**0.005**
Insulin (*µU/ml*) ^*^	19. 10(13.80–27.30)	17.70(13.00–22.10)	-	0.107
HOMA-IR^*^	4.01(3.21–6.21)	3.72(2.71–5.33)	-	0.139

TC, total cholesterol; TG, triglyceride; HDL, high density cholesterol; LDL, low density cholesterol; FSG, fasting serum glucose; ALT, alanine amino transferase; AST, aspartate amino transferase; MDA, malondialdehyde; TAC, total antioxidants, HOMA-IR, homeostasis model assessment insulin resistance.

† P-value for TG, Insulin and HOMA-IR based on Mann-Withney; otherwise based on independent T-test using equal variable.

* TG, Insulin and HOMA-IR are presented based on median (P25–P75) and other variables data are presented based on mean (SD).

**Table 3.** Genotypes and allele distribution of −276 G>T gene polymorphism of adiponectin in study population

**Polymorphism**	**NAFLD (n =75) n *(%)***	**Control (n =76) n *(%)***	**OR ^††^ (95% CI)**	**p ^†^**
**Genotype**				
GG	33 (44.0)	39(51.3)	1	
GT	32(42.7)	28 (36.8)	1.40(−8.75 to 4.99)	0.489
TT	10(13.3)	9(11. 8)	1.44(0.69 to 2.81)	0.344
**+276 G>T**				
allele				
G	98(65.3)	52(34.7)	1.00	0.321
T	106(69.7)	46(30.3)	1.28(0.78 to 2.10)	

† P-value based on Chi-Square Tests.

†† Odds Ratio (OR) based on logistic regression analysis adjusted for age and gender (p>0.05).

As shown in table 4, ALT level in patients with NAFLD was significantly higher than control group in all three genotypes (p=0.022); meanwhile AST level was higher in GT and GG genotypes in NAFLD group as compared to control (p<0.05). TAC level in TT genotype was significantly lower in patients as compared to healthy subjects with the same genotype (p<0.05), (Table 4).

**Table 4.** The comparison of study Anthropometric indices and biochemical parameters according to +276 G>T polymorphism of Adiponectin between study group

**Variable**	**Genotype**	**NAFLD (n=75) mean (SD)**	**Control (n=76) mean (SD)**	**Mean Difference (95% CI)**	**p ^††^**
**BMI (*kg/m*^2^)**					
	GG	32.18(3.19)	28.52(2.28)	2.76(−0.16 to 5.54)	0.060
	GT	31.95(4.48)	32.52(4.42)	−0.12(−2.47 to 2.22)	0.917
	TT	31.51(4.21)	31.23(3.78)	0.26(−1.53 to 2.04)	0.773
**WHR**					
	GG	0.92(0.07)	0.89(0.05)	0.02(−0.04 to 0.08)	0.436
	GT	0.92(0.05)	0.89(0.07)	0.03(4.04 to 0.07)	0.050
	TT	0.91(0.06)	0.90(0.05)	0.01(−0.01 to 0.04)	0.398
**WC (*cm*)**					
	GG	104.77(11.13)	96.08(6.33)	8.68(−0.23 to 17.59)	0.056
	GT	103.36(8.92)	101.41(9.21)	1.95(−2.73 to 6.65)	0.408
	TT	102.40(9.67)	100.14(8.74)	2.22(−2.10 to 6.55)	0.309
**Cholesterol (*mg/dl*)**					
	GG	192.20(28.70)	172.22(28.21)	30.58 (3.91 to 57.26)	**0.027**
	GT	184.28(41.42)	192.25(29.26)	−6.42 (−25.92 to 13.07)	0.512
	TT	179.96(34.91)	188.51(28.27)	−10.20 (−25.43 to 5.03)	0.186
**HDL (*mg/dl*)**					
	GG	43.20(8.62)	44.55(11.40)	−2.72(−13.64 to 8.19)	0.603
	GT	44.37(10.24)	48.42(11.55)	−3.31(−9.05 to 2.42)	0.252
	TT	42.15(13.26)	49.05(11.82)	−5.66(−11.17 to 0.15)	**0.044**
**LDL (*mg/dl*)**					
	GG	113.16(28.59)	95.33(26.68)	25.48(−3.11 to 54.08)	0.077
	GT	109.05(38.26)	114.32(28.26)	−3.90(−22.19 to 14.38)	0.671
	TT	97.95(32.19)	113.24(24.30)	−15.85(−29.52 to −2.19)	**0.024**
**ALT (*U/L*)**					
	GG	46.90(27.69)	32.77(8.81)	24.67(7.75 to 41.56)	**0.007**
	GT	50.75(25.88)	28.57(9.59)	20.64(10.18 to 31.10)	**<0.001**
	TT	50.12(26.26)	24.23(9.56)	26.03(18.04 to 34.02)	**<0.001**
**AST (*U/L*)**					
	GG	34.39(17.7)	22.82(5.63)	11.67(5.88 to 17.45)	**<0.001**
	GT	33.03(12.57)	23.00(7.28)	9.88(4.30 to −15.47)	**0.001**
	TT	28.2 (13.79)	24.4(4.41)	8.11(−1.5 to 17.74)	0.092
**FSG (*mg/dl*)**					
	GG	87.70(12.66)	90.22(9.09)	−3.94(−15.66 to 7.78)	0.485
	GT	91.21(9.01)	90.96(9.83)	0.13 (−4.86 to 5.12)	0.959
	TT	90.84(12.85)	89.17(10.36)	1.49(−4.12 to 7.11)	0.598
TG (*mg/dl*) ^*^					
	GG	136.50(125.25–240.50)	150.00(102.00–217.50)	-	0.814
	GT	151.50(109.00–182.50)	116.50(92.25–208.75)	-	0.772
	TT	171.00(117.50–236.50)	108.00(67.00–195.00)	-	0.166
**MDA (*nmol/ml*)**					
	GG	2.69 (1.01)	3.11(1.44)	0.78(−0.96 to 0.80)	0.467
	GT	2.90 (1.19)	26.46 (0.78)	0.78(−0.96 to 0.80)	0.098
	TT	2.78(0.83)	2.87(1.05)	−0.005(−0.33 to 0.34)	0.677
**TAC (*mg/dl*)**					
	GG	1.42(0.29)	1.56(0.30)	0.78(−0.96 to 0.80)	0.061
	GT	1.50(0.23)	1.58(0.23)	−0.05(−0.36 to 0.26)	0.225
	TT	1.38(0.22)	1.66(0.26)	−0.16(−0.24 to −0.07)	0.022*
Insulin (*µU/ml*) ^*^					
	GG	18.90(15.37–25.82)	16.10(12.55–48.24)	-	0.513
	GT	18.10(13.55–26.95)	18.45(13.82–25.85)	-	0.941
	TT	19.70(14.00–29.15)	17.50(11.90–21.20)	-	0.188
HOMA-IR^*^					
	GG	3.91(3.11–6.57)	3.73(2.69–11.00)	-	0.814
	GT	3.94(2.91–6.07)	4.34(2.94–5.83)	-	0.772
	TT	4.29(3.25–6.38)	3.62(2.54–4.51)	-	0.166

BMI, body mass index; WC, waist circumference; WHR, waist to hip ratio; FSG, fasting serum glucose; MDA, malondialdehyde; TAC, total antioxidants, HOMA-IR, homeostasis model assessment insulin resistance.

†† P-value based on ANCOVA adjusted for age and gender.

* TG, Insulin and HOMA-IR are presented based on median (P25 –P75) and other variables data are presented based on mean (SD).

Mean dietary intake including energy, protein, fat, vitamins C and E have not shown any significant differences between NAFLD and control group (p>0.05), (Table 5).

**Table 5.** Comparison of energy, macro and micronutrient intakes between study groups

**Variable**	**NAFLD (n=75) mean (SD)**	**Control (n=76) mean (SD)**	**Mean Difference (95%CI)**	p ^*^
**Calories (kcal)**	2815.06 (536.35)	2794.93(448.64)	−20.13(−179.29 to 139.03)	0.803
**Protein (*g/day*)**	93.88 (21.62)	88.88 (16.60)	−4.99(−11.20 to 1.20)	0.113
**Carbohydrate (*g/day*)**	437.38 (89.72)	438.49(79.58)	1.10(−26.18 to 28.39)	0.936
**Total fat (*g/day*)**	89.70 (27.03)	91.52(24.35)	1.82(−6.45 to 10.10)	0.664
**Vitamin E (*mg/day*)**	14.08 (5.60)	14.63(4.11)	0.80(−1.03 to 2.12)	0.496
**Vitamin C (*mg/day*)**	127.14(66.09)	140.59(64.18)	13.44(−7.50 to 34.39)	0.207

* P-value of variables based on independent T-test using equal variable.

The comparison of dietary intakes according to +276 G>T adiponectin gene polymorphism between case and control groups is presented in table 6. Patients with GT genotype have significantly lower fat consumption and vitamin E intake as compared to control group with the same genotype (p<0.05). No significant difference was observed for other nutrients according to the genotypes of −276 G>T adiponectin gene polymorphism (p>0.05).

## Discussion

In the present study, we evaluated the possible association between +276G>T adiponectin gene polymorphism, metabolic parameters and nutritional intake among the Iranian NAFLD population. To our review of literature, this is the first report, which evaluates the association between different genotypes of +276 G/T polymorphism in adiponectin gene with metabolic parameters among the Iranian NAFLD patients. Our results showed no significant association between (+276 G/T) polymorphism and risk of NAFLD in the studied groups. Several studies have evaluated the effect of gene polymorphism of adiponectin gene on NAFLD in different populations ^4,20,23,24^.

Musso *et al* ^23^ found an association between +45 T/G and +276 G/T polymorphisms of adiponectin gene and risk of NAFLD in their study; however, their features included non-obese and normo lipidemic subjects. In another case-control study by Zhou *et al* ^24^, the G/T variant at +276 may decrease the susceptibility to NAFLD. In the line of our study, Wong *et al* ^4^ found no association between polymorphism of adiponectin at position +276 and NAFLD in Chinese patients. Also, in Japanese population different genotypes of adiponectin +276 G/T polymorphism did not show significant difference between NAFLD patients and control group ^20^.

Insulin resistance and central obesity are well-known characteristic associated with intra-abdominal fat accumulation which has been positively correlated with liver fat ^25^. In our study, insulin resistance and HOMA-IR did not show significant association with different genotypes of +276 G/T polymorphism in NAFLD patients; these findings were also supported by previous studies ^26,27^. However, these results were not consistent with Melistas *et al* ^18^ study and the mentioned parameters were significant according to genotype of TT in +276 G/T gene polymorphism. It can be explained that the subjects in cross-sectional study by Melistas *et al* ^18^ included non-diabetic women without any other disease.

In this study we aimed to evaluate the environmental impact, along with genetic factors in the outbreak of NAFLD. As we showed in table 6, normal samples with TT genotype had low calorie, fat and high vitamins E and C consumption (although this difference was not significant due to small sample size). So, we can say that individuals with mutant and pathogen genotype which have proper diet, can overcome the disease. But to evaluate any additional impact we should study this issue in larger sample sizes. In addition, we observed that NAFLD patients with GT genotype consumed small amounts of vitamin E in their usual diet. In this regard, Musso *et al* ^27^ reported a low dietary ascorbic acid and tocopherol intake in patients with non-alcoholoic steatohepatitis. Also, Erhardt *et al* ^28^ reported reduced tocopherol intake as dietary antioxidant compounds in patients with NASH compared to healthy group. In fact, oxidative stress is one of the most common factors involved in the pathogenesis of NAFLD; moreover vitamins C and E are well-known antioxidants capable in blocking distribution of radical reactions. These antioxidants play important roles in histological improvement of inflammation in NAFLD ^29^.

On the other hand, hepatic *de novo* lipogenesis can be affected by dietary macronutrients; high fat intake reduced hepatic *de novo* lipogenesis in obese hyper insulinemic subjects compared to obese normo insulinemic subjects ^30^. Therefore, dietary recommendation should be based on individual status ^4^ and even genetic background ^31^. It is clear that dietary modification is the easiest and even the most efficient way to reduce risk factors of chronic disease ^32^.

## Conclusion

Overall, among the metabolic parameters, TAC in TT genotype was significantly lower, AST in GT, GG genotypes, and ALT in all three genotypes were higher in NAFLD patients as compared to healthy subjects. Also non-statistical significance of other results might be attributed to the difference in the stage of disease; on the other hand, this is the first study to compare nutritional intakes according to adiponectin +276G/T gene polymorphism in patients with NAFLD.

One limitation of this study was its relatively small sample size. Therefore, further studies with larger sample size and interventional designs are needed to confirm the effect of dietary compounds in different +276 G/T genotypes in nonalcoholic fatty liver disease.
